# Supplementary material for: Are carbon emissions trading and green financial instruments synergistic? -Comprehensive quantitative research based on content analysis
Source: PLoS One. 2024 Mar 7;19(3):e0298601. doi: 10.1371/journal.pone.0298601 (PMC10919601; doi:10.1371/journal.pone.0298601)
Supplement: S1 Data — (ZIP) [file pone.0298601.s001.zip › Data/地级市Z_F工作报告.zip!/╣π╢1⁄2/╤⌠╜¡╩╨/└┤╫╘╣1⁄2╓┌║┼í╛┬φ┐╦╩2╛▌═°í┐_2008.docx]

各位代表：

　　现在，我代表市人民政府向大会作政府工作报告，请予审议，并请政协各位委员和其他列席人员提出意见。

2007年工作回顾

　　一年来，市人民政府在中共广东省委、省人民政府和中共阳江市委的正确领导下，在市人大及其常委会和市政协的监督支持下，坚持以邓小平理论和“三个代表”重要思想为指导，以科学发展观统领经济社会发展全局，认真贯彻落实党的十七大和省第十次党代会精神，积极实施“工业立市、旅游强市、商贸旺市、农业稳市、科教兴市”五大战略，以工业为主攻方向，以招商引资为突破口，大力发展临港工业和区域特色经济，着力打造广东电力能源基地和面向西南的物流配送基地，全市国民经济保持健康平稳较快发展，社会各项事业全面进步，市五届人大一次会议确定的各项任务顺利完成。全市完成生产总值395.8亿元，增长14.2%。人均生产总值16878元，增长13.3%。全社会固定资产投资134.7亿元，增长31.5%。社会消费品零售总额222.1亿元，增长18.8%。固定资产投资和社会消费品零售总额增速均创近十年新高。地方财政一般预算收入12.97亿元，增长24.64%，增幅居全省第14位；财政总收入36.94亿元，财政总支出36.42亿元，收支保持平衡。金融机构本外币存款余额318.3亿元、贷款余额136.7亿元，分别比上年末增长12%和27.4%。

　　——能源基地建设全面提速，发展后劲明显增强。阳江核电站核岛负挖工程去年9月动工，各项工程建设进展顺利。广东华厦阳西电厂去年6月正式浇灌第一方混凝土，首台机组将于2009年6月商业运行并网发电。阳江抽水蓄能电站完成了可行性研究和进场道路建设，各项前期工作顺利推进。雅图（阳江）风电设备生产基地项目、阳江首个500千伏蝶岭输变电站先后动工。其他电力能源项目的各项前期工作进展顺利。

　　——园区建设成效显著，临港工业发展加快。工业园区建设势头良好。与珠三角地区共建5个产业转移工业园，其中4个被认定为省级产业转移工业园，投入开发建设资金8.9亿元，签订入园协议、意向项目78个，总投资79亿元，建成投产项目14个。临港工业不断发展。全智立体图书高级印刷品生产基地等一批工业项目先后上马建设，嘉吉公司成功重组丰源粮油公司并增资扩产，龙尚鞋业、德恩富光盘等项目建设加快。全市完成工业总产值492.6亿元，增长20.6%；其中规模以上工业总产值339.8亿元，增长25.3%。

　　——利用外资大幅提升，内外源型经济协调发展。全市实际利用外商直接投资1.3亿美元，增长23.3%。外贸出口总额10.6亿美元,增长8.8%。民营经济保持快速发展，全市民营企业与个体工商户53853户，比上年增加9020户；规模以上民营工业完成总产值199.6亿元，增长25.3%，全市民营经济实现税收18.2亿元，增长22%。

　　——产业结构进一步优化，自主创新能力持续提高。三大产业全面发展，产业布局和结构日趋合理，三大产业比例由上年的25.8∶38.2∶36调整为24.1∶40.7∶35.2。工业高级化趋势进一步增强，产业集中度不断提高。农业基础地位更加稳固，全市农业总产值150.8亿元，增长5.5%。第三产业持续发展，连锁经营、物流配送、批发市场等现代流通业发展势头良好。文化、体育、医疗保健、餐饮等服务业取得长足发展。第三产业实现增加值139.5亿元，增长13.8%。自主创新能力持续提高，新增2家国家级、15家省级高新技术企业，2家省级民营科技企业，1家省级工程研发中心和1家省级农业科技创新中心，1个中国驰名商标；建成了3个省火炬计划特色产业基地、4个省级专业镇；阳东五金刀剪科技产业化基地被认定为省部产学研结合示范基地；专利申请量达935件，增长16.1%，继续保持粤西地区领先地位；市和阳东县分别被评为全国科技进步先进市和先进县。

　　——农业经济健康发展，新农村建设稳步推进。农业结构调整成效明显，粮经比例由上年的44∶56调整为42∶58。畜牧业稳定发展，全市肉类总产量13.7万吨，增长10.6%。林业取得新成绩，全市完成造林面积11.2万亩，造林质量居全省前列。农业产业化持续加快，现有省级农业龙头企业5家，市级农业龙头企业13家。农产品质量安全监管力度不断加大，蔬菜、水果的质量检测合格率分别达90%和95%，阳西县近江牡蛎（程村蚝）标准化吊养示范基地、平冈农场水产养殖场被定为农业部水产健康养殖示范基地，3个省级无公害标准化示范区在建，12个产品通过国家无公害农产品认证，6个产品通过国家绿色食品认证。农村基础设施建设和社会事业发展取得新成绩。完成灌区干支渠清淤维修162公里，培训农村劳动力2.5万人，农村富余劳动力实现转移就业5万人。生态文明村建设深入开展，农村生产生活条件明显改善。

　　——特色经济发展势头良好，县域经济日益壮大。旅游业更加繁荣兴旺。旅游景区景点和配套设施建设进一步加快，涛景度假村、碧桂园凤凰酒店、东湖酒店先后建成开业。旅游宣传促销力度加大，开通了阳江旅游目的地营销系统“阳江旅游”网站。创建旅游品牌工作取得新成绩，阳春市被命名为“中国优秀旅游城市”。2007年，全市接待游客535.2万人次，旅游总收入28亿元，分别增长13.5%和13.1%。海洋渔业加快发展。海洋捕捞业结构继续优化，外向型水产养殖业蓬勃发展，水产品深加工能力不断提高，水产品出口势头良好，渔港升级建设全面开展。成功举办第五届南海（阳江）开渔节。全市水产品总产量100.1万吨，渔业总产值57.9亿元，分别增长5%和7.5%。水产品总产量、人均产量等指标继续居全省地级市前列。南药生产加工有新发展。全年南药种植面积18.2万亩，增长28.6%。南药总产值5亿元，增长25.9%。五金刀剪业持续发展。成功举办了第六届中国（阳江）国际刀剪博览会暨第七届阳江市旅游文化节。全市五金刀剪业总产值126亿元，增长18%。县域经济呈现加快发展态势，各县(市、区)规模以上工业增加值、固定资产投资、社会消费品零售总额等主要经济指标均实现较快增长。

　　——基础设施建设不断加快，城乡面貌进一步改善。交通设施建设掀起新热潮。省道113线阳春南段一级公路二期建设基本完成，省道369线春湾至罗阳段二级公路、开阳高速公路阳西立交及县城连接线进入全面施工阶段，阳阳铁路阳江港码头专用线、开阳高速公路阳江立交及连接线南段已进场施工，阳江港8号泊位3.5万吨级通用码头建成运营，疏港大道建设基本完工。云阳高速公路阳江至阳春段完成设计招投标。沿海高速公路联络线白沙至程村段二级公路等项目前期工作顺利推进。电网建设实现新突破。全年共有20个项目开工，9个项目投产，完成投资7.5亿元，建成35千伏及以上输电线路67.76千米，增加变电容量20万千伏安，是建市以来最大规模的电网建设。城市建设日新月异。江台路、文明路、马曹路中段、马南路中段、新江东路西侧等一批城市道路建设和改造工程竣工通车，漠江水厂三期扩建工程顺利投产，城乡防灾减灾工程建设加快推进。第三届房地产展销会成功举办。城市管理继续加强，开展了市区市容市貌百日整治大行动，城市面貌不断改观。

　　——各项改革取得新进展，发展活力持续增强。国有企业改革稳步推进，市汽运集团成功实现改制重组，华龙集团破产重整工作顺利推进。银行业机构改革稳步实施，成立市银行业协会，在全省地级市率先成立邮政储蓄银行分支机构，农信社改革步伐进一步加快，停业整顿城信社市场退出工作取得显著成效。中小企业融资方式不断创新，信贷两难问题初步得到解决。规范市直机关公务员津贴补贴工作有序推进。环境保护和交通领域综合行政执法改革深入开展。水利工程、兽医管理、粮食流通和种子经营等各项体制改革不断加快。

　　 ——环境保护卓有成效，生态环境保持良好。认真实施环境规划，推行建设项目环保准入，从源头上防止污染产生。治污保洁工程有效推进，市第一净水厂扩建工程基本建成，城镇生活污水处理厂建设加快。生态示范村创建工作积极开展，全市创建各级生态示范村42个。环境质量不断改善，空气质量达到国家一级标准，漠阳江饮用水源水质达到国家Ⅱ类标准。节能减排成效明显，化学需氧量（COD）和二氧化硫排放量均在省下达的总量控制指标以内，单位生产总值能耗、单位工业增加值能耗分别下降4%和8%，环境保护责任制考核连续四年被省评为优秀。节约集约用地成效明显，连续九年实现耕地占补平衡，土地利用效率进一步提高，确保了重大项目的用地需要。

　　——社会建设全面加强，文明程度不断提高。教育发展取得新成果。九年义务教育进一步巩固和提高，全市实施免费义务教育学生达30.2万人。农村义务教育经费保障政策落实到位，C、D级危房彻底消除。高中阶段教育发展较快，阳春一中高中部新校区等新建学校建成招生，市技工学校晋升为省级重点学校，全市高中阶段教育在校生比上年增加8166人。高等教育稳步推进，阳江职业技术学院办学质量逐年提高。弱势群体教育得到关注和重视，市特殊教育学校建成招生。教育信息化建设加快，教育教学水平不断提高，高考成绩取得较大进步。公共卫生应变控制力有效提高，疾控预警与处置机制逐步建立，卫生人才培养力度不断加强。中医工作取得新进展，阳春中医院被省中医药局授予首批中医名院。结核病、艾滋病和人禽流感等重大疫病得到有效防治。医疗急救指挥体系进一步完善，初步建立了市120急救指挥中心。无偿献血工作连续4次获全国先进城市称号。“红十字会”体制有效理顺。文化事业健康发展。文化名城建设“五个十工程”有序推进，广东海上丝绸之路博物馆建设顺利，“南海I号”古沉船整体打捞出水进馆，冯盎将军陵园迁建和关山月故居修建项目正在抓紧实施。基层文化阵地建设不断加强，文化活动丰富多彩，文艺创作繁荣兴旺，《开渔之歌》等一批文艺作品荣获省精神文明建设“五个一工程”优秀奖等奖励。文化市场整治取得新成效，文化市场秩序进一步规范。文物和非物质文化遗产保护工作扎实推进。声像档案工作取得突破性进展。体育设施建设稳步推进，群众性体育运动广泛开展，成功举办了市第三届运动会、市第八届体育节、中国（阳江）国际风筝会和承办2007年广东省青少年帆板赛。人口和计划生育工作水平不断提高，低生育水平趋于稳定，年度人口自然增长率为 6.9‰。婚育新风进万家活动、层级动态管理责任制、计生技术队伍建设等工作走在全国前列，被确定为全国婚育新风进万家活动示范市、全国计划生育保险试点市。国防动员、兵员征集、民兵预备役和双拥工作进一步加强，再次被评为全国双拥模范城。民族宗教、妇女儿童、外事侨务、广播电视、民政、统计、人防、打私、气象、地震、市志、爱卫等各项工作均取得新成绩。

　　——民生工程取得较大进展，和谐阳江建设取得新成效。以“一保五难”为重点的各项民生工程全面实施，和谐阳江建设稳步推进。城镇新增就业岗位3.3万个，城镇登记失业率3％。覆盖城乡居民的社会保障体系不断完善。参加社会保险81.6万人，增长7.9%。纳入低保救济31971户74440人，享受五保待遇12987人。新型农村合作医疗覆盖率达83.9％，新农合制度覆盖全市所有行政村，初步解决了农村群众看病难问题。完成镇通村委会公路硬底化360公里，新开通村客运班线7条，全市客车通村率达75%，解决了48个行政村群众的出行难问题。完成32宗农村饮水工程，解决了23个镇125条自然村32.8万人的饮水难问题。经济适用住房和廉租住房建设不断加强，全面解决了2003年底登记在册的337户特困户的住房问题；完成农村贫困户泥砖住房改造3249户，转产转业水上渔民上岸安居142户，缓解了困难群众住房难问题。人民群众的生活水平不断提高，城镇居民年人均可支配收入11116元，增长8.9%；农村居民年人均纯收入4838元，增长5.5%。社会治安综合治理成效显著，打黑除恶专项斗争取得重大成果，群众安全感进一步增强。安全生产和消防工作明显加强，安全事故四项指标全面下降，重特大安全生产事故得到有效遏制。产品质量和食品安全专项整治深入开展，食品药品安全监督管理取得初步成效。价格服务进万家活动有序推进，行政收费进一步规范，涉企降槛减负工作有效落实。市场价格监管逐步加强，居民消费价格总指数涨幅控制在2.9%，低于全省0.8个百分点，价格总水平基本保持稳定。市场经济秩序继续好转，打击非法传销、制假售假等违法活动取得明显成效。人民调解和信访工作不断加强，群体性事件明显减少，各种人民内部矛盾纠纷得到认真调处和化解，社会更加和谐稳定。

　　——精神文明和民主法制建设稳步推进，政府自身建设不断加强。党的十七大精神学习宣传和解放思想学习讨论活动深入开展，群众性精神文明创建活动稳步推进，沙扒等7个社区被评为广东省首批“六好”平安和谐社区。与民主党派、工商联、无党派人士和工、青、妇等人民团体的联系更加密切，科学民主决策机制进一步完善。办复省、市人大代表议案及建议135件、政协委员提案128件。办理行政复议案件33宗。普法教育、法律援助、劳动教养和刑释解教人员安置帮教工作有新发展。政府自身建设不断加强，科学行政、民主行政、依法行政水平进一步提高。政务公开制度全面推进，建成了市级电子政务外网平台和行政审批电子监察系统，阳春市被评为全国政务公开先进县和示范点。法制、审计、监察和廉政建设继续加强，违法违纪案件得到严肃查处，损害群众利益的不正之风得到有效纠正。

　　各位代表，过去一年，是我市坚持科学发展，促进社会和谐取得显著成效的一年。这些成绩的取得，是在省委、省政府和市委的正确领导下，全市人民齐心协力、奋力拼搏的结果。在此，我谨代表市人民政府，向全市广大工人、农民、知识分子、干部职工，向驻阳江人民解放军、武警官兵、人民警察和各民主党派、人民团体、社会各界人士致以崇高的敬意！向长期关心支持我市发展的港澳台同胞、海外侨胞以及国际友人表示衷心的感谢！

　　在肯定成绩的同时，我们也清醒地看到，我市经济社会发展还存在不少困难和问题。主要是：经济总量小，结构不合理；科技创新能力弱，产业竞争力不强；农民增收难度大，部分低收入群体生活比较困难；食品药品安全、医疗卫生、就业社保等方面工作仍需不断加强；城市规划、建设、管理的水平有待进一步提升；一些干部的思想观念、工作作风还不完全适应科学发展观的要求，运用市场经济手段和法律手段解决问题的能力有待进一步提高等。对此，我们必须采取更加有力的措施认真加以解决。

2008年工作安排

　　2008年是全面贯彻落实党的十七大精神的重要一年，也是阳江建市20周年。做好今年的各项工作，意义重大，任务艰巨。政府工作的总体要求是：高举中国特色社会主义伟大旗帜，坚持以邓小平理论和“三个代表”重要思想为指导，深入贯彻落实科学发展观，继续解放思想，坚持改革开放，紧紧围绕发展与和谐两大主题，积极实施“工业立市、旅游强市、商贸旺市、农业稳市、科教兴市”五大战略，以工业为主攻方向，以招商引资为突破口，调整优化经济结构，提高经济增长质量，加强节能减排和生态环境保护，努力改善民生和促进社会和谐，全力推进经济社会又好又快发展。

　　2008年主要预期目标是：生产总值增长13%，万元GDP能耗下降3.5%，主要污染物排放总量控制在省下达我市指标以内，工业总产值增长20%，农业总产值增长5%，全社会固定资产投资增长23%，社会消费品零售总额增长15%，外贸出口总额增长10%，实际利用外商直接投资增长20%，地方财政一般预算收入增长15%，居民消费价格总水平涨幅控制在3.5%以内，城镇登记失业率控制在3.5%以内，人口自然增长率控制在7.2‰以下，城镇居民人均可支配收入增长9%，农村居民人均纯收入增长6%左右。

　　按照上述总体要求，实现全年目标和任务，必须进一步解放思想，把解放思想贯穿到各项具体工作的始终，以战略的眼光和思维分析形势、制定政策、谋划举措、推进发展，争当欠发达地区实践科学发展观的排头兵。从我市实际出发，要切实做到三个“更加注重”：一是更加注重壮大经济总量和提高发展质量。千方百计提高发展速度，做大经济总量，尽快缩小与发达地区的差距，同时坚持好字优先，加快调整产业结构，努力提高经济发展的质量和效益，推动经济社会又好又快发展。二是更加注重环境保护。既要努力优化投资环境，降低投资门槛，大力招商引资，又要走新型工业化路子，切实加强节能减排和生态环境保护，绝不能以牺牲环境为代价，走高污染、高能耗、低效益或先污染后治理的老路。三是更加注重改善民生。既要加快经济发展步伐，又要注重改善民生，提高人民群众生活水平，维护好人民群众的合法权益，促进社会和谐稳定，做到“见物又见人”，让全市人民共享改革发展的成果。

　　具体要做好以下工作：

一、全力推动工业发展提速增效

　　强势推进电力项目建设。力争国家批准阳江核电项目9月份正式动工，首期建设6台100万千瓦级机组。加快推进广东华厦阳西电厂、雅图（阳江）风电设备生产基地、蝶岭500千伏输变电工程等在建电力能源项目建设。继续抓好阳江抽水蓄能电站、海陵风电场等重大能源项目前期工作。积极发展电力能源配套产业，延伸产业链条，加快产业集聚。

　　积极培育壮大临港工业。加强阳江港区的规划建设，以阳江港为依托，积极引进大型企业集团和重大工业项目，打造临港工业核心区。加快推进德恩富光盘生产基地、龙尚鞋业、全智立体图书高级印刷生产基地等一批在建工业项目建设，做好上海三盛集团阳西丰头船舶修造基地和阳江港物流保税中心等重大项目的跟踪服务工作，带动装备制造、电子信息、海洋生物、汽车配件、钢铁等行业的发展，推动工业适度重型化。

　　大力扶持重点企业发展。积极实施扶优扶强政策措施，培育发展一批主业突出、带动能力强、支撑作用大的企业。支持嘉吉粮油等一批重点企业做大做强。大力实施名牌带动战略，鼓励企业争创国家和省名牌产品、驰名和著名商标，培育一批名牌企业。建立完善中小企业服务体系，积极推进全民创业，鼓励和扶持民营企业开展制度、技术、管理创新，增强综合竞争力，努力提升民营经济发展水平。

　　加快完善工业园区建设。继续加强高新区等工业园区的规划建设工作，相对集中地推进园区的成片开发，不断完善基础设施。把好园区项目准入关，建立园区项目建设用地优先保障机制、准入机制和退出机制，提高投资强度和投资密度，打造集经济、技术、环保、绿色于一体的品牌园区。积极承接珠三角等经济发达地区产业转移，做好签约项目的跟踪落实，年内争取一批项目在各类园区动工建设，一批项目建成投产，形成新的经济增长点。

二、扎实推进社会主义新农村建设

　　着力构筑现代农业产业体系。全面落实支农惠农政策，增强农业和农村经济发展活力。继续调整优化农业结构，在稳定粮食生产的基础上，扩大优质特色经济作物种植规模，提高农业效益，促进农民增收。积极发展南药种植加工业，突出抓好春砂仁、益智、广藿香等南药的种植和加工。积极发展现代渔业，做大水产养殖业，精心组织实施阳西县国家级和阳东县省级渔业科技入户工程；做优海洋捕捞业，努力开发外海、深海和远洋渔业资源；做强水产品加工与流通业，重点扶持谊林海达、顺兴、万事达等渔业企业发展壮大；加强渔港建设，认真做好闸坡中心渔港一期工程验收和二期工程申报工作，加快沙扒国家一级渔港建设，落实东平国家级中心渔港建设项目。办好第六届南海（阳江）开渔节。发展现代林业，充分发挥林业在建设生态文明中的作用。优化畜牧业结构，继续落实能繁母猪补贴政策，提高生猪生产能力。

　　加快农业产业化进程。积极扶持农业龙头企业，培育壮大一片天制药集团等龙头企业。推进“万村千乡”市场建设工程，加快农产品流通体系建设。完善农业科技创新和服务体系建设，加快农业先进技术推广应用，推进农业机械化。加快无公害产品、绿色食品、有机食品以及地理标志保护产品基地建设，推动农业标准化生产，努力提高农产品质量安全水平。完善动植物疫病防控体系建设，加强动植物疫情防控，做好高致病性禽流感等重大动植物疫病的防控工作，确保人民群众吃上放心肉、放心菜。

　　进一步改善农业生产条件。积极实施农田水利基本建设人大议案，着力加强农业基础设施建设。加快东湖、合水、岗美等水库和台平三丫联围除险加固等水利工程建设，提高农业抗灾能力。加强排灌渠系建设，改善农田排涝和灌溉条件。加快田间道路运输系统建设，为机械化耕作创造条件。加强农业综合开发土地治理，搞好地力培肥，提高单位耕地产出能力。帮助和指导农民和养殖户做好冻灾后生产工作，尽量降低受灾损失。

　　大力发展农村各项事业。推进新农村规划编制工作，抓好村庄试点，逐步改善农村人居环境。扎实推进农村合作医疗工作，逐步完善合作医疗运行机制，解决好特困群众的医疗救助问题。继续加强镇通村委会公路硬底化建设，增设农村客运候车亭，增开农村客运班车，方便农民出行。组织实施好渔民转产转业项目，努力完成阳春、江城和阳西水上居渔民安居工程。加大农村贫困户泥砖住房改造力度，切实改善农村困难群众住房难问题。继续推进农村饮水工程，保障农村饮用水安全。加强农村劳动力技能培训，提高农村劳动力素质。做好村级组织换届选举工作。

三、着力提高开放型经济发展水平

　　大力开展招商引资。强化投资服务管理中心行政服务功能，构建大行政服务平台，大力改善政务环境。进一步清理行政事业性收费，降低营商成本。完善招商引资政策，强化奖惩措施。优化口岸通关环境，突出抓好阳江港集装箱运输定期航班工作。大力开展产业招商，结合我市资源优势和产业优势，积极引导外资投向临港工业、文化旅游业、农海产品深加工及现代服务业。创新招商引资方式方法，加快建立以企业为主体的招商机制，提高招商引资实效。积极做好签约外资项目的服务工作，力促一批签约项目动工建设。

　　努力扩大外贸出口。优化出口产品结构。大力开发适合国际市场的新产品，提高产品出口竞争力。着力做强做大板材生产加工、水产品深加工等出口企业，扩大家具、园艺、畜牧、水产等优势产品出口。引导企业强化品牌意识，推进 “阳江刀剪”国际出口区域品牌创建工作，培育更多出口品牌企业和龙头产品。加大出口市场的开拓力度，优化出口市场结构。积极应对国际贸易摩擦，适应国家外经贸政策的调整，促进外经贸稳定增长。

四、积极提升自主创新能力

　　加快科技创新平台建设。强化企业的技术创新主体地位，鼓励企业建立工程技术研究开发中心，争取年内培育2家省级工程技术中心。进一步完善市五金刀剪创新中心“5中心1数据库”的建设，加快市五金刀剪模具研发中心的建设步伐，积极创建国家五金刀剪产品质量检验检测中心，强化生产力促进中心、科技企业孵化器等各类创新服务平台的服务功能，为中小企业的创新发展提供服务。加快发展专业镇，争取年内培育3个省级专业镇。

大力改造提升传统产业。鼓励创建技术中心，积极运用电子信息、生物技术、新材料、节能环保等高新技术改造提升传统产业。实施五金刀剪和食品加工等传统产业提升工程，引导企业加强技术创新。创建国家级火炬计划五金刀剪特色产业基地。依靠科技进步推动社会主义新农村建设，加强农业科技攻关和先进实用技术推广，全面铺开农村信息直通车工程。

　　扎实推进产学研结合。鼓励企业围绕产业技术需求，与高校科研院所组建“产学研”战略联盟，重点抓好五金刀剪省部“产学研”战略联盟建设和六大“产学研”研发或产业化基地建设。组织举办部属高校、重点科研院所与我市企业的对接活动，促进企业与高校的交流合作。

　　加强知识产权和科普工作。扎实推进省级知识产权试点城市工作。加强知识产权宣传教育，在中小学开展知识产权教育试点工作，积极引导企业培育品牌、提升品牌、经营品牌、延伸品牌，重点扶持、发展和保护一批拥有自主知识产权的名牌产品，培育发展一批知识产权优势企业。充分发挥五金刀剪行业协会的自律作用，加强刀剪知识产权保护。

五、大力发展第三产业

　　着力提升旅游业发展水平。认真实施《阳江市旅游总体发展规划》，加快旅游景区景点建设，重点抓好三山岛国际旅游度假区游艇和风帆项目、阳春温泉度假村等一批旅游项目的建设。努力打响中国优秀旅游城市、中国温泉之乡等旅游品牌，加强“南海I号”的旅游宣传推介和营销策划，开发“南海I号”古船模型等系列旅游产品，策划、拓展海上丝绸之路博物馆旅游项目。大力开展旅游招商，打造新的旅游亮点。

　　加快发展现代物流业。加快推进粤西烟草配送中心、粮油批发配送中心、交通物流中心等项目的规划建设，大力发展连锁经营、物流配送、电子商务等现代流通业，逐步把我市建设成区域性物流基地，提升第三产业发展水平。

　　积极扩大内需。加快消费市场设施建设，优化消费环境，扩大消费规模。大力发展金融、保险、会计、信息、设计装饰和法律服务等现代服务业，引导形成汽车、房地产、娱乐、休闲、体育健身等消费热点，推动消费结构升级。

六、努力加强生态环境保护和城乡建设管理

　　继续推进绿色阳江建设。深化环境保护责任制考核，推行污染减排一票否决和环境责任追究制。开展全市第一次污染源普查。加强水源保护和大气污染防治，推进漠阳江综合整治和治污保洁工程，强化机动车尾气污染防治。加快推进生活污水和垃圾处理设施建设，抓好医疗垃圾等危险废物安全处理。重视节能减排工作，加强对全市32家重点用能单位的节能管理和服务。抓好建设项目环境管理，认真把好环保准入关。积极发展环保科技和环保产业，大力推进循环经济和清洁生产，推广应用新型墙体材料，进一步促进建筑节能工作。保护好农村的生态环境，广泛开展环境优美乡镇和生态示范村等创建活动。加强海洋环境管理和保护，积极开展人工鱼礁和海洋与水产自然保护区建设，实施好阳西、阳东、江城国家级和海陵省级海域使用管理百强县示范活动及东亚海岸带综合管理平行示范点项目。严格执行土地管理制度，积极推进节约集约用地。大力清理闲置土地，盘活土地资源。加强政府土地一级市场建设，健全土地储备交易运作机制，提高土地利用效率和效益。

　　强化城乡建设管理。力争云阳高速阳江至阳春段年底动工建设，加快开阳高速公路阳江立交及连接线、阳茂高速织立交至阳西县城连接线、阳江港铁路专用线、省道369线阳春春湾至罗阳段改建工程、白沙至程村二级公路、那龙至核电三级公路、阳江港11-14号及19号泊位等项目建设，不断完善交通基础设施。大力推进城南新区、核电科研后勤生活区、一河两岸和开阳高速公路连接线等城建重点区域的规划建设工作。加快完善北湖公园、鸳鸯湖公园等公园的规划和配套设施建设，改善市民休闲活动场所。抓好瑞禾路、北环路等旧城改造项目，推进高凉路、三环路和漠江中路的拆建工程。狠抓市区防洪工程首期、阳春城市防洪工程等4宗城市防洪工程建设。加强城市规划，尽快制定城区各项控制性详细规划，强化城市管理，推进城市公用设施建设，美化城市环境。推进以中心镇为重点的城镇建设，全面完成12个中心镇的总体规划编制工作。

七、继续深化各项改革

　　深化国有企业改革。加强国有资产监管，确保国有资产保值增值。按照成熟一个、改革一个的原则，稳步推进国有企业改革，加快劣势企业退出市场。妥善处理好国企改革的遗留问题，认真做好改制企业职工安置工作，维护职工的合法权益。

　　完善财税管理机制。健全各县（市、区）异地招商税收分成制度，理顺市内税收征管关系。大力开展税收宣传，优化税收征管模式。按照公共财政的要求，继续推进部门预算、国库集中支付、政府采购、收支两条线等各项改革，提高各级理财水平，做到生财有道、聚财有方、用财有规，确保财政稳健运行。加强财政资金审计，强化财政资金管理。

　　抓好行政审批制度改革。缩减行政审批事项，规范备案制度，简化审批程序，缩短审批时限，强化审批责任，把行政审批纳入规范化、制度化轨道。

　　稳步推进农村信用社、投融资体制改革，努力加快农业、科技、教育、文化、体育、卫生和地方公路养护等各项改革。

八、加快推进社会建设

　　在加快经济发展的同时，更加注重社会建设，着力保障和改善民生，努力使全市人民学有所教、劳有所得、病有所医、老有所养、住有所居。

　　优先发展教育。巩固提高九年义务教育水平，实现全市城乡免费义务教育，积极推进县域义务教育均衡发展。加快发展普通高中教育，扩建21所中学。大力发展中等职业技术教育，支持市技工学校申报国家级技校，扩建市第一职业高级中学。完善阳江职业技术学院办学条件，努力办好高等教育。规范和促进民办教育发展，探索外来工子女受教育的有效办法。加快完善市特殊学校。加强师资队伍建设，积极实施基础教育“十百千人才工程”，培养一批名校长和名教师，不断提高教育教学水平。

　　积极扩大就业。大力宣传贯彻实施《就业促进法》，促进以创业带动就业，争取全市新增就业岗位3.4万个，转移农村劳动力5.6万人。深入实施再就业扶持政策，着力解决就业困难群体就业，促进下岗失业人员再就业。做好城镇新增劳动力、大学生和残疾人的就业工作。加快县（区）级劳动力市场建设。加强职业技能培训工作。落实国家对农民工的政策，依法维护劳动者权益。

　　完善社会保障体系。制订和实施城镇居民医疗保险办法和困难企业退休职工参加基本医疗保险办法。继续推进被征地农民养老保险制度试点工作，加快推进全市被征地农民养老保险工作。修订出台《阳江市职工生育保险暂行办法》，扩大生育保险覆盖面。完善失业和工伤保险制度，健全失业保险与促进就业联动机制。狠抓扩面征缴工作，加强社保基金监管，确保基金安全。

　　全面发展文化卫生体育等社会事业。推进文化体制改革，繁荣文化事业，发展文化产业。加快广东海上丝绸之路博物馆建设，切实做好“南海I号”古沉船和出水文物的保护。着力推进文化名城“五个十工程”，提升公共文化服务能力，强化文化市场监管。加快发展广播影视、新闻出版等事业，全面实施农村电影放映工程和广播电视“村村通”工程，丰富群众文化娱乐生活。加强人才市场建设，提高人才资源市场化配置服务水平，切实抓好人才的引进培养工作。完善公共卫生和医疗服务体系。加强农村卫生管理体制改革。推进社区卫生服务工作。健全农村药品供应和监督网，加强药品流通监管，确保药品安全。推进中医药振兴工作，建设市中医院新院。切实抓好艾滋病、狂犬病、登革热等重大传染病的防治，做好职业病防治工作。加大卫生技术人才培养力度。加强计划生育管理，突出治理出生人口性别比偏高问题，稳定低生育水平。积极发展体育事业，深入开展全民健身运动，逐步完善体育设施，建设市体育运动学校和市水上活动中心，发展体育竞技项目，积极备战国内外重要体育赛事。加强国防建设、民兵预备役和“双拥”工作，继续开展争创全省双拥模范城活动。抓好《阳江市志》的出版发行。推进档案馆舍和档案资源建设。进一步做好外事侨务、民族宗教、打私、人防、气象、统计、老龄和妇女儿童及残疾人等工作，推进社会各项事业全面发展。

九、注重维护社会和谐稳定

　　加强产品质量和食品安全监管。深入开展产品质量和食品安全专项整治，建立和完善产品质量安全监管网络，加强检验检测等公共服务平台和产品质量标准体系建设，构建保障产品质量安全的长效机制。

　　加强平安阳江建设。健全完善突发公共事件应急处置机制，不断提高应对突发公共事件能力。加强社会治安综合治理，不断完善社会治安防控体系。继续保持严打高压态势，依法打击各种犯罪活动。大力扫除“毒赌黄”，净化社会风气。坚持以铁的手腕抓好安全生产，落实政府安全生产责任制，强化企业主体责任，加强安全生产专项整治，强力推进“一畅两会”消防专项整治和重大火灾隐患政府挂牌督办工作，坚决防范和遏制重特大事故。加强信访工作，建立健全基层信访调处网络，规范信访秩序，预防和妥善处置群体性事件。深入开展“五五”普法，加强法律援助，做好刑满释放人员帮教和社区矫正工作。

　　加强市场价格监管。认真落实临时价格干预措施，强化市场价格监管调控，坚决打击各种哄抬价格、扰乱市场价格秩序的不法行为，严格控制出台政府提价项目，加强对价格的宣传引导，千方百计保持价格总水平和市场价格秩序基本稳定。

　　做好改善民生工作。从人民最关心、最直接、最现实的利益问题入手，加大民生工作力度。进一步做好城乡低保、农村“五保”供养、城乡特困户生活与医疗救助等经常性的社会救助工作。推进农村敬老院建设，抓好第三批敬老院的改建扩建。逐步提高五保供养水平，使全市五保集中供养标准达到每人每月200元以上，分散供养标准达到每人每月150元以上。发展慈善事业。加大对红十字事业的扶持力度，充分发挥各级红十字会的作用。办好市慈善会，指导协助各县（市、区）成立慈善机构。加强经济适用房、普通商品房和廉租房建设，向社会供应一批经济适用住房、廉租住房，进一步缓解城镇低收入家庭住房难问题。

十、切实加强政府自身建设

　　加快政府职能转变和管理方式创新，推进科学行政、民主行政、依法行政，努力建设行为规范、运转协调、公正透明、廉洁高效、群众满意的服务型政府。

　　当好解放思想的表率。加强学习，以宽阔的胸怀和开放的视野，虚心学习发达地区和兄弟市的好经验、好做法。加快转变思想观念，不断拿出改革开放的新举措，创出改革开放的新模式，走出改革开放的新路子。要努力寻找影响发展的思想障碍和机制体制的不足，以思想大解放促进经济大发展，以思想大解放促进和谐社会建设，以思想的大解放促进政府管理的改革和创新，抢抓机遇，实现阳江跨越式发展。

　　当好科学发展的表率。以科学发展观统领经济社会发展全局，坚持以人为本，围绕人的幸福来谋划发展。充分运用市场机制、经济规律办事，提高整体发展效率和质量。对涉及经济社会发展的重大决策，主动征询各方面意见，力求科学缜密，避免决策失误。综合考虑不同区域的资源环境承受能力、开发密度和发展潜力，组织开展主体功能区规划方案编制工作，积极谋划符合主体功能区规划的重点产业和重点项目，加紧研究承接珠三角新一轮产业转移的工作方案，争取更多的大项目、好项目转移落户阳江。

　　当好高效服务的表率。以强烈的使命感和责任心推动阳江加快发展，把精力集中到抓工作上来，集中到干事创业上来。增强服务意识，创新服务方式，全力提高行政效能和服务质量。健全和完善抓落实的工作机制，做到办事高效、运转协调、政令畅通。

　　当好依法行政的表率。自觉接受人大及其常委会的监督和政协的民主监督，积极听取各民主党派、工商联、无党派人士和人民团体的意见，认真办理人大代表、政协委员的议案、建议和提案，提高办理质量。加强电子政务建设，推进政务公开。规范行政执法行为，强化行政执法监督，提高依法行政水平。

　　当好务实为民的表率。把改善民生作为政府工作的出发点和落脚点，切实做到群众不受益的政策不出台，群众不支持的措施不实施，损害群众利益的人和事不迁就，真心真意办好一批事关群众切身利益、真正惠及百姓的实事，让人民群众充分享受改革开放和现代化建设的成果。

　　当好廉洁勤政的表率。大力弘扬胡锦涛总书记倡导的八个方面良好风气，筑牢各级干部拒腐防变的思想防线。切实加强廉政建设，贯彻落实党风廉政建设责任制，健全监督制约机制。深入开展反腐败斗争，严肃查处违纪违法案件。开展民主评议活动，切实纠正损害群众利益的不正之风，努力打造风清气正、公平透明、廉洁高效的政务环境。

　　各位代表！阳江已经站在新的发展起点上，形势催人奋进，新的征程任重道远。我们一定要高举中国特色社会主义伟大旗帜，坚持以邓小平理论和“三个代表”重要思想为指导，全面落实科学发展观，继续解放思想，坚持改革开放，在省委、省政府和市委的正确领导下，团结和带领全市人民，以只争朝夕的精神，坚韧不拔的毅力，改革创新的锐气，埋头苦干，奋勇前进，为全面完成今年各项工作任务，为推动阳江实现科学发展、社会和谐而努力奋斗！
